# Using Steady-State Visual Evoked Potentials to Characterize Wide-Ranging Retinopathy Linked to *CRB1*: Implications for Clinical Trials

**DOI:** 10.34133/csbj.0042

**Published:** 2026-04-30

**Authors:** Kim Eliane Stäubli, Marc Pabst, Roni O. Maimon-Mor, Ana Catalina Rodriguez-Martinez, H. Steven Scholte, Mariya Moosajee, Tessa Marlijn Dekker

**Affiliations:** ^1^ Institute of Ophthalmology, University College London, London EC1V 9EL, UK.; ^2^ Experimental Psychology, Division of Psychology and Language Sciences, University College London, London WC1H 0AP, UK.; ^3^ Moorfields Eye Hospital NHS Foundation Trust, London EC1V 2PD, UK.; ^4^ Great Ormond Street Hospital for Children NHS Foundation Trust, London WC1N 3JH, UK.; ^5^ Department of Psychology, University of Amsterdam, 1018 WT Amsterdam, Netherlands.; ^6^ The Francis Crick Institute, London NW1 1AT, UK.

## Abstract

Rapid advances in gene therapy are moving sight rescue treatments for inherited retinal diseases (IRDs) within reach, creating an urgent need to understand how these conditions affect neural signaling from the eye to the brain. However, capturing functional change across the diverse IRD sight-loss spectrum using a unified testing framework is challenging. Computational neuroimaging may help address this gap by exploiting known principles of visual-system tuning to derive more sensitive and computationally meaningful markers of visual function. This is particularly important in *CRB1* retinopathy, an IRD with a strikingly wide phenotype range, where neural impacts across the full disease spectrum are not yet characterized. To investigate the functional impact of *CRB1* retinopathy, we recorded steady-state visual evoked potentials (ssVEPs) in 72 eyes from 18 patients and 18 sighted controls using a patient-friendly, large-field protocol embedding phase-reversing sinusoidal gratings and full-screen flashes into age-appropriate videos. Fitting these data with neural tuning functions revealed significant ssVEP attenuation in patients, with the greatest reductions in those with generalized retinal degeneration, alongside shifts in spatial-frequency tuning toward lower frequencies. Our results show that with appropriate stimulus selection, ssVEPs offer a reliable response-free measure of visual function that correlates well with behavioral vision assessments and discriminates between *CRB1* subgroups, with the optimally sensitive stimulus for detecting functional variation varying with the level of vision. Computational electroencephalogram-based neuroimaging thus provides quantitative insights across the wide spectrum of *CRB1* pathology and represents a valuable complementary tool for evaluating disease progression and treatment outcomes in *CRB1* retinopathy and related IRDs.

## Graphical Abstract

**Figure:**
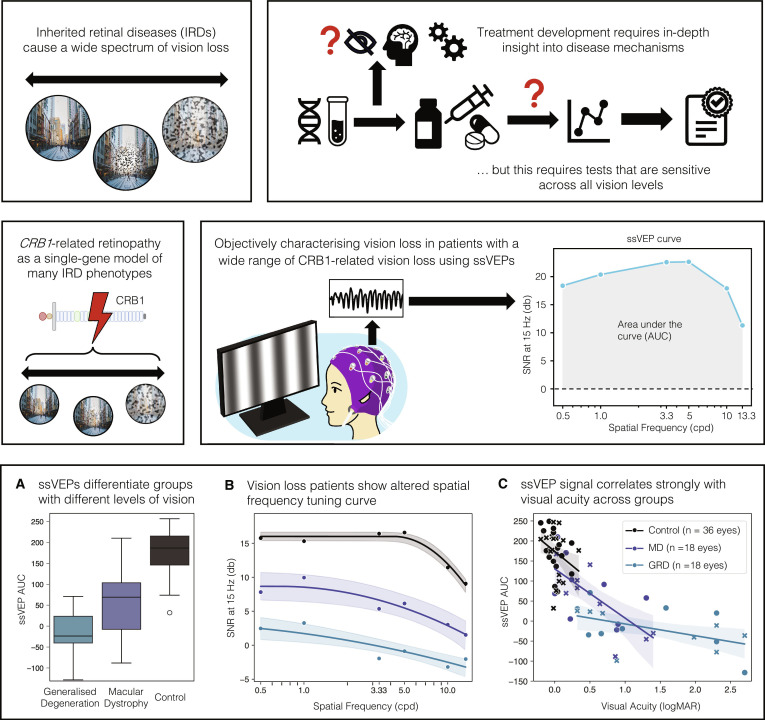

## Introduction

Understanding how changes to the genetic code alter structure and function across different biological scales, from cells to neural networks to perception and action, has been a major challenge in modern biomedical research. Inherited retinal diseases (IRDs), a leading cause of vision loss in children and the working-age population [1,2], illustrate this problem: While disease-causing genetic variants are increasingly well characterized, linking them to systems-level functional signatures is still in its early stages. Modern computational models, informed by knowledge about how the visual system processes information, can help bridge this gap by offering sensitive, quantitative, and mechanistically interpretable indices of visual system function. In this study, we apply computational techniques that integrate information about brain responses to a range of visual stimuli to model the neural signatures of IRD-related vision loss and how they relate to clinical measures of function.

This is now more relevant than ever. Owing to the eye’s unique position as an accessible part of the central nervous system, IRDs are currently at the forefront of advances in gene therapy and neuronal intervention. With the emergence of new sight rescue therapies, there is a pressing need to comprehensively characterize these genetically and clinically heterogeneous conditions and find ways to measure how potentially rescued signals can be transmitted to and processed by the brain after treatment.

*CRB1*-related retinopathy is an IRD associated with a particularly broad spectrum of phenotypes. For this reason, it offers a single-gene model to examine how common IRD vision loss profiles are reflected in neural function. *CRB1*-related retinopathy is caused by biallelic mutations in the *Crumbs cell polarity complex component 1* (*CRB1*, Online Mendelian Inheritance in Man #604210), a gene primarily expressed in retinal photoreceptor and Müller glia cells [3,4]. *CRB1* plays a critical role in retinal development and the long-term preservation of retinal integrity [5], with mutations affecting retinal lamination and leading to varying spatiotemporal patterns of photoreceptor degeneration [4]. The most prevalent and severe forms of *CRB1*-related retinopathy include Leber congenital amaurosis (LCA) and early-onset severe retinal dystrophy (EOSRD), where patients present with severe vision loss in infancy or early childhood, which may worsen with age [5,6]. Other phenotypes include macular dystrophy (MD), cone-rod dystrophy (CRD), and retinitis pigmentosa (RP), which typically present during adolescence or early adulthood. While CRD and RP manifest with cone- or rod-specific symptoms that become more generalized with disease progression, MD is characterized by localized degeneration at the macula, leaving the peripheral retina largely unaffected.

Despite substantial progress made in understanding the molecular functions and pathophysiology of *CRB1* [7,8], we know little about the impact of *CRB1* mutations on the postretinal visual system. While there is currently no cure for *CRB1*-related retinopathies, gene-therapy research is progressing rapidly [9–11]. To inform effective translation of these treatments to clinical benefit, we need to comprehensively characterize how *CRB1* mutations alter visual processing and develop clinical trial endpoints that can reliably and objectively capture changes in visual function across the disease spectrum.

However, the applicability of traditional visual function assessments varies considerably across visual impairment severities and is limited in young children. As a result, recent *CRB1* natural history studies have encountered challenges in obtaining consistent clinical measures across the entire cohort [12,13], often yielding incomplete datasets [6], excluding patients with insufficient data [14], or restricting samples to those with better vision [15]. Moreover, many visual function tests rely on patients’ verbal articulation, button presses, or observable behaviors like looking or pointing, making them susceptible to attentional lapses and response- and evaluator-induced biases.

Visual evoked potentials (VEPs) provide an objective, low-demand alternative for assessing visual function directly at the visual cortex, with a well-established history of use in both clinical and research settings [16]. Steady-state VEPs (ssVEPs), in particular, are elicited by the sustained, rapid, and periodic modulation of a visual stimulus attribute [17], which elicits cortical responses carrying rhythms of that same frequency in the electroencephalogram (EEG)—provided the visual system is capable of processing the changing stimulus attribute [18]. ssVEP-based assessments hence provide a response-free, brain-based quantification of visual function and offer insight into eye-to-brain signaling and neural processing signatures of different vision loss conditions. Despite their potential, VEPs have not been systematically assessed in *CRB1*-related retinopathies and questions remain about their applicability in patients with very low vision due to challenges with fixation and sustained attention to hardly visible stimuli [19].

In this study, we aimed to characterize ssVEP response profiles in *CRB1*-related retinopathy and address key challenges of objectively assessing visual function in wide-ranging impairment by using an adapted ssVEP protocol combining 4 key elements. First, we integrated pattern and full-field flickering stimuli within a single protocol to capture visual responses sensitively and reliably across disease severity levels. Second, we used an extra-large-field stimulation setup to ensure constant retinal stimulation even in patients with poor central fixation. Third, we employed computational modeling grounded in known principles of visual spatial tuning [20] to improve the mechanistic interpretability of our measure, inspired by similar developments in other neuroimaging modalities [21,22]. Specifically, we extended traditional electrophysiological metrics by modeling ssVEP response curves across multiple spatial frequencies, allowing us to examine how *CRB1* mutations affect retinocortical spatial-frequency tuning. This approach allows pathological changes in ssVEP signal to be interpreted not only as a set of isolated responses but as a parametric representation of cortical spatial filtering, characterized by interpretable parameters like preferred spatial frequency, response gain, and bandwidth. It also provides an informative summary measure of overall neural responsiveness. Finally, by modeling responses at the group level while controlling for within-subject correlation, we extend prior analyses of visual function that typically focus on single eyes and inter-eye averages or overlook such dependencies.

Our analysis revealed *CRB1*-related ssVEP attenuation and shifts in spatial-frequency tuning toward lower frequencies. Our ssVEP-derived metrics distinguished between macula-only and generalized retinal degeneration and closely tracked behavioral vision measures. They also showed good reliability overall, although the optimally informative stimulus type varied by vision level. These findings demonstrate that computational modeling of large-field ssVEPs including a range of stimuli can provide objective, clinically relevant measures that offer mechanistic insights across the wide spectrum of *CRB1* pathology. This could be a valuable complementary tool for evaluating disease progression and novel treatment outcomes.

## Methods

### Participants

**Fig. 1.**
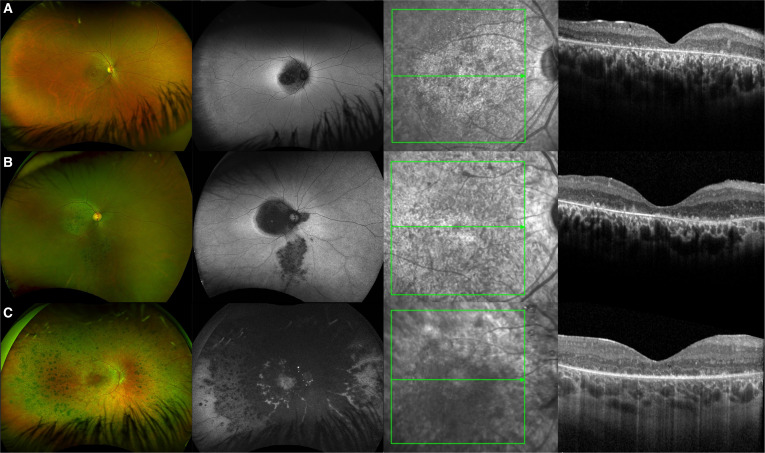
Widefield color fundus photographs, fundus autofluorescence (FAF), and optical coherence tomography (OCT) images illustrating the phenotypic spectrum of *CRB1* retinopathy. (A) Right eye of a 34-year-old female patient with *CRB1*-related macular dystrophy (MD) revealing hypo-autofluorescence (AF) in the posterior pole with a surrounding hyper-AF ring. Spectral domain (SD)-OCT depict a coarse, thickened, and disorganized retina with outer nuclear layer (ONL) and ellipsoid zone loss. Electroretinography (ERG) indicated marked bilateral central macular dysfunction, with preserved generalized cone responses. Best-corrected visual acuity (BCVA) at the time of testing was 0.86 logMAR. (B) Right eye of a 34-year-old male *CRB1* cone-rod dystrophy (CRD) patient, demonstrating macular-to-peripheral reginal degeneration and BCVA of 0.88 logMAR with ERG results confirming generalized cone and rod dysfunction. SD-OCT shows a thickened retina with ONL and ellipsoid zone loss. (C) Right eye of an 18-year-old female patient with *CRB1*-related early-onset severe retinal dystrophy (EOSRD) with a BCVA of 0.5 logMAR showing peripheral nummular pigmentation, preserved perivascular retinal pigment epithelium, and generalized retinal degeneration seen as hypo-AF in the FAF image sparing the macular region as seen with preserved AF signal in the foveal region. SD-OCT depicts a coarse, thickened, and disorganized retina with ONL loss of ellipsoid zone and foveal hypoplasia grade 1B.

Eighteen patients with *CRB1*-related retinopathy, aged 15 to 58 (*M* ± SD: 36.17 ± 14.61 y; 6 females), were recruited from Moorfields Eye Hospital, London, UK. All carried molecularly confirmed biallelic (pathogenic or likely pathogenic) variants in the *CRB1* gene, as determined through genetic testing and variant interpretation performed clinically and in research settings, as previously described [3,15,23]. Nine patients presented with MD, 6 with LCA/EOSRD, and 3 with the CRD phenotype. Examples of typical retinal findings in the included phenotypes, as observed on color fundus photographs, fundus autofluorescence, and optical coherence tomography scans, are provided in Fig. 1. Visual acuity, measured as the logarithm of the minimum angle of resolution (logMAR), ranged from 0.00 logMAR to perception of light (PL) (*M* ± SD: 0.97 ± 0.81 logMAR), age at onset ranged from 0.1 to 40 years (*M* ± SD: 15.87 ± 12.58 y), and disease duration ranged from 3 to 49 years (*M* ± SD: 20.29 ± 12.13 y). Sixteen patients were of White and 2 were of Asian or Asian British ethnic background. Demographic characteristics for individual patients are reported in Table 1. Eighteen individuals aged 21 to 54 years (*M* ± SD: 31.13 ± 10.93 y; 11 females) were included as a sighted control benchmark. Their best-corrected visual acuity ranged from −0.2 to 0.34 logMAR (*M* ± SD: 0.01 ± 0.12). Controls and patients did not significantly differ in their age or gender, but *CRB1* patients had significantly lower visual acuity than controls (*P* < 0.001). For group comparisons, the *CRB1* cohort was divided into 2 equally sized subgroups, defined by the extent of retinal involvement: one characterized by localized MD and the other by generalized retinal degeneration involving both rods and cones (see Results).

**Table 1.** Patient demographics. The methodology of genetic testing and variant interpretation has been described previously [3,15,23]. All variants were classified as pathogenic or likely pathogenic, and all cases of compound heterozygosity were confirmed to be in *trans* during genetic testing.

ID	Age (y)	Sex	VA OS (logMAR)	VA OD (logMAR)	Phenotype	Disease duration (y)	Zygosity	Variant 1 cDNA (variant 1 protein)	Variant 2 cDNA (variant 2 protein)
002	48.1	F	0.22	0.16	MD	18.1	Compound heterozygous	c.1696G>T (p.Glu556Ter)	c.498_506del (p.IIe167_Gly169del)
003	16.6	F	0.48	0.32	LCA/EOSRD	16.0	Compound heterozygous	c.2548G>A (p.Gly850Ser)	c.4006-10A>G
004	15.6	M	0.70	0.50	MD	3.6	Compound heterozygous	c.498_506del (p.Ile167_Gly169del)	c.1576C>T (p.Arg525*)
005	17.5	F	0.50	0.50	LCA/EOSRD	13.5	Compound heterozygous	c.455G>A (p.Cys152Tyr)	c.3014A>T (p.Asp1005Val)
006	52.2	M	0.24	0.32	MD	12.2	Compound heterozygous	c.2401A>T (p.Lys801*)	c.498_506del (p.Ile167_Gly169del)
007	39.1	M	0.00	0.64	MD	9.1	Compound heterozygous	c.498_506del (p.Ile167_Gly169del)	c.4142C>G (p.Pro1381Arg)
008	17.4	M	0.04	0.02	MD	9.4	Compound heterozygous	c.498_506del (p.Ile167_Gly169del)	c.2308G>T (p.Gly770Cys)
009	29.5	M	0.16	0.32	MD	11.5	Compound heterozygous	c.498_506del (p.IIe167_Gly169del)	c.3827_3828del (p.Glu1276Valfs*4)
011	40.5	M	0.32	1.4	CRD	20.5	Compound heterozygous	c.498_506del (p.IIe167_Gly169del)	c.1431delG (p.Ser478Profs*24)
012	34.3	F	0.88	0.86	MD	9.3	Compound heterozygous	c.2290C>T (p.Arg764Cys)	c.498_506del (p.Ile167_Gly169del)
013	34.0	M	0.96	0.88	CRD	28.0	Compound heterozygous	c.470G>C p.Cys157Ser	c.2506C>A (p.Pro836Thr)
014	58.9	F	1.30	1.30	MD	32.0	Compound heterozygous	c.3991C>T (p.Arg1331Cys)	c.4142C>T (p. Pro1381Leu)
015	16.2	M	1.58	1.98	LCA/EOSRD	16.1	Compound heterozygous	c.2401A>T (p.K810*)	c.2872delAG (p.S958fs)
016	54.1	M	HM	PL	LCA/EOSRD	49.1	Homozygous	c.1831T>C (p.Ser611Pro)	
017	46.1	M	PL	HM	LCA/EOSRD	40.1	Homozygous	c.1831T>C (p.Ser611Pro)	
018	34.1	M	HM	HM	LCA/EOSRD	33.1	Homozygous	c.2843G>A (p.Cys948Tyr)	
019	48.2	F	0.7	0.7	CRD	18.2	Homozygous	c.2639A>G (p.Ans880Ser)	
020	48.5	M	0.9	1.4	MD	24.5	Compound heterozygous	c.498_506del (p.Ile167_Gly169del)	c.584G>T (p.Cys195Phe)

CRD, cone-rod dystrophy; EOSRD, early-onset severe retinal dystrophy; F, female; LCA, Leber congenital amaurosis; M, male; MD, macular dystrophy; OD, oculus dexter (right eye); OS, oculus sinister (left eye); VA, visual acuity

Participants with a history of neurological disorders or injuries, including epilepsy or seizures, as well as those with EEG contraindications were excluded from the study. Two additional sighted controls were tested but not included in the present report as they met the criteria for visual impairment according to World Health Organization guidelines [24] (visual acuity in the better eye worse than 0.30 logMAR).

Testing was conducted at Moorfields Eye Hospital, London, in line with the principles outlined in the Declaration of Helsinki. Ethical approval was provided by the Moorfields Eye Hospital National Health Service Foundations Trust and the National Research Ethics Committee (Research Ethics Number: 12/LO/0141) and by the UCL Research Ethics Committee (Project ID Number: 4846/002), and all participants provided written informed consent prior to participation, which included consent for the acquisition and publication of medical images. For participants under 18 years of age, written parental or legal guardian consent was further obtained prior to participation.

### Visual acuity and peripheral visual field testing

All participants underwent visual acuity testing using Early Treatment Diabetic Retinopathy Study charts and/or assessment for light perception, the current gold-standard measures of visual acuity, prior to their ssVEP assessment (except 1 typically sighted control). A subset of patients (*n* = 14) further underwent kinetic perimetry testing that evaluated their peripheral visual field boundary as part of a concurrent study [12,15], conducted with the Octopus 900 perimeter (The Haag-Streit Group, Köniz, CH). The peripheral visual field boundary was determined by systematically moving small light stimuli from the outer edge of the individual’s visual field inward and the individual indicating via button presses when they first perceived the stimuli [25]. The stimuli included II4e, IV4e, and V4e Goldmann targets, which are standardized spots of light with 0.2°, 1.7°, and 1.7° diameter, respectively, presented at maximum intensity.

### ssVEP assessment: stimuli and paradigm

Participants were seated in a dimly lit room 83 cm from the screen (LCD monitor, 60 Hz refresh rate; 380 cd/m^2^ maximum luminance; gamma calibrated; field of view: 45.7° × 26.7°) and were presented with phase-reversing sinusoidal gratings, displayed at 15 reversals per second embedded within age-appropriate videos. Audio was delivered via 2 speakers placed behind the left and right edges of the screen. Stimuli were created using PsychoPy [26] and pyglet [27] in Python v.3.8.17 (Python Software Foundation, Wilmington, DE, USA). A neutral density filter (Stage Depot; effective neutral density filtering 1.3) was placed in front of the monitor, reducing the overall luminance to minimize discomfort in light-sensitive subjects, with a maximum luminance of 20 cd/m^2^ at the observer’s position. Total testing time was approximately 1.5 h per participant, including setup and breaks.

**Fig. 2.**
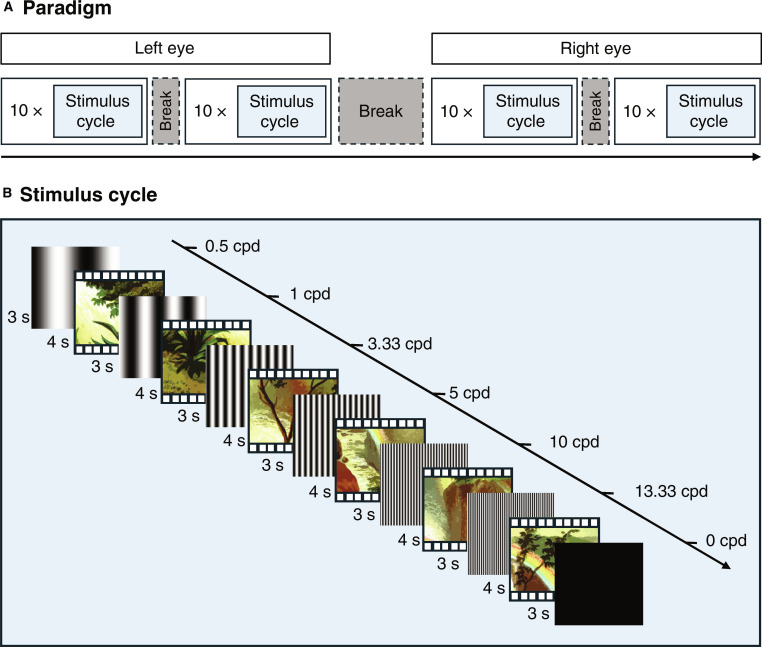
Illustration of stimulus presentation. (A) Testing procedure. Participants completed 20 stimulus cycles with each eye, interspersed with breaks. (B) A stimulus cycle. During each stimulus cycle, 7 contrast-reversing vertical gratings were presented in 3-s intervals at 15 reversals per second, interleaved with a continuously playing movie. Gratings were presented monocularly and in ascending order of spatial frequency across a range of 0.5 to 13.33 cycles per degree (cpd), followed by a full-screen flicker (0 cpd). The movie audio was played throughout the whole session, and movie segments (duration jittered between 3 and 5 s, with an average of 4 s) were shown in between steady-state visual evoked potential (ssVEP) stimulus presentations.

Visual stimuli were presented monocularly, at full contrast (effective Michelson contrast calculated as 99.9% close to the screen and 99.6% at the observer’s distance), and in ascending order of spatial frequency across 0.5 to 13.33 cycles per degree (cpd), with a full-screen flashing stimulus (0 cpd) at the end of each stimulation cycle. Embedding stimuli with a spatial component alongside full-screen flicker stimuli within a single paradigm enabled us to directly compare ssVEPs across conditions to identify the most effective stimulus features for our population. Each spatial frequency was displayed for 3 s followed by a movie presentation lasting 3 to 5 s (duration jittered to reduce predictability). For each eye, participants completed 20 stimulus cycles, divided into 2 runs of 10 cycles each, that were separated by a short rest break. During stimulus presentation, the movie visuals were replaced by the overlaid stimuli, but participants could hear the movie audio throughout the session, enabling them to follow the narrative. Figure 2 provides an illustration of the testing procedure. The paradigm was framed as a game where participants pressed a button whenever they detected a coin sound within the movie audio while observing the ssVEP stimuli on the screen. This was done to evaluate the feasibility of gamifying the ssVEP task to engage young participants and those with severe visual impairment.

Scalp EEG was recorded with a 32-channel system at a sampling rate of 2,048 Hz (Biosemi Active V2; Biosemi B.V., Amsterdam, NL), with electrodes positioned according to the standard 10–20 system locations. Electrode offsets were kept below 50 mV. As standard eye-tracking calibration is challenging for most of our patients due to fixation difficulties, we placed electrodes around the eyes and on the earlobe to capture ocular activity instead of using conventional eye tracking. While these data were not included in our current analysis, they present a valuable opportunity for future investigation.

### Data analysis

#### Preprocessing

**Fig. 3.**
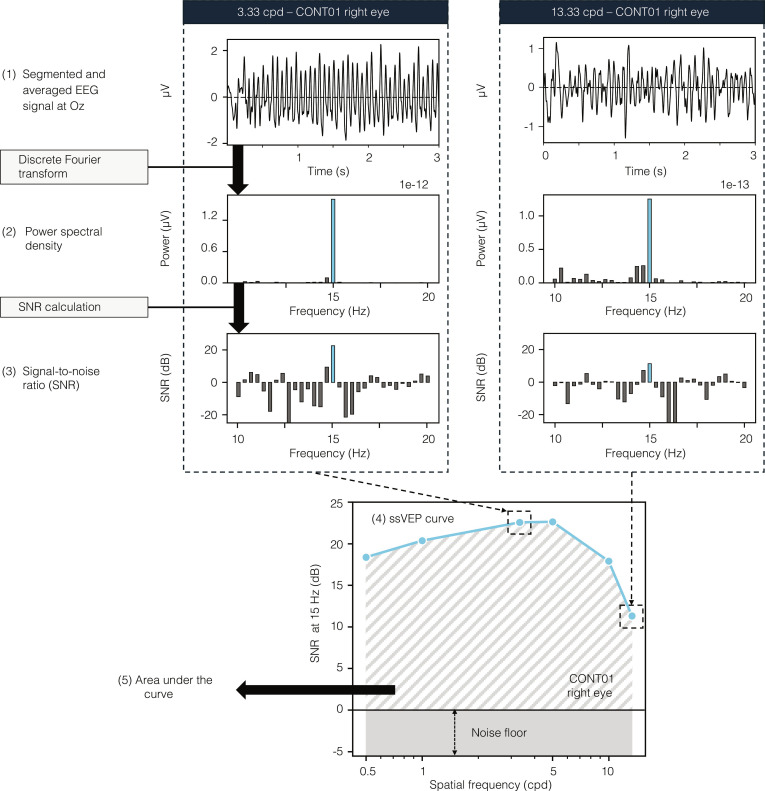
Illustration of the computation of individual ssVEP curves. Electrical signals recorded from a central occipital electrode (Oz) are referenced to the average of 2 adjacent electrodes (O1 and O2), segmented into individual epochs, and averaged across the tested eye and stimulus condition (1). EEG, electroencephalogram. Fast Fourier transform is applied to calculate the power spectral density (PSD) over the 3-s stimulation period within each averaged epoch (2). The signal-to-noise ratio (SNR) is derived from the PSD spectrum by dividing each frequency bin by the average of its 2 neighboring frequency bins to either side, excluding immediate neighbors, and applying a log transformation on the result. The SNR at the phase-reversal frequency (15 Hz, indicated in light blue) is analyzed as an indicator of the visual system’s sensitivity to the presented stimulus (3). SNR values across all pattern-reversal stimuli for each eye constitute the ssVEP curve. Values below 0 indicate that the signal falls below the noise floor, making it indistinguishable from noise. (4) The area under the curve (AUC) is calculated from the ssVEP curve using the trapezoidal rule for each eye (5).

EEG data were preprocessed with the MNE-Python toolbox v1.4.2 [28] using Python v3.11.0.4 (Python Software Foundation). ssVEPs of each eye were treated as distinct data points in all statistical analyses to account for differences in visual function between 2 eyes of the same participants. Figure 3 provides an overview of the EEG data analysis pipeline.

Electrical signals recorded from a central occipital electrode (Oz) were referenced to the average of 2 adjacent electrodes (O1 and O2), an approach often referred to as a local one-dimensional Laplacian montage in line with the International Society for Clinical Electrophysiology of Vision guidelines [19,29]. The data were band-pass filtered offline between 4 and 40 Hz using a finite impulse response filter, segmented into individual epochs, and averaged across stimulus condition within each tested eye. All 20 epochs per eye and condition were included in the averages to maintain an equal number of segments across participants. A fast Fourier transform was then applied to calculate the power spectral density over the 3-s stimulation period within each averaged epoch.

Signal-to-noise ratio (SNR) was derived from the power spectral density spectrum for all epochs and frequency bins by dividing each frequency bin by the average of its 2 neighboring frequency bins to either side, excluding immediate neighbors. The resulting SNR values were logarithmically converted to decibels to account for distribution skewness common in ratio data such as SNRs [30]. In line with standard protocols [19], the SNR measure at the phase-reversal frequency (15 Hz) was subsequently analyzed as an indicator of the of sensitivity to the presented stimulus:

$${\mathrm{SNR}}_{15\;\mathrm{Hz}}=10\cdot \log_{10}\frac{m_{15\;\mathrm{Hz}}}{\left(m_{14\;\mathrm{Hz}}+m_{14\bar{.3}\;\mathrm{Hz}}+m_{15.\bar{6}\;\mathrm{Hz}}+m_{16\;\mathrm{Hz}}\right)/4}\;\mathrm{dB}$$ (1)

Visualizing the resulting SNR values across individual spatial frequencies for a given eye provides an overview of the responsiveness profile of the eye’s retinocortical pathways as reflected in the ssVEP (ssVEP curves). To derive a summary measure of the ssVEP curve that can be related to other measures of visual function, we computed the area under the curve (AUC) using the trapezoidal rule [31]. The AUC quantifies the total area between the ssVEP curve and the noise threshold (SNR = 0 dB) across the 0.5-to-13.33-cpd range and thus offers insights into overall neural responsiveness to pattern stimuli without relying on assumptions about curve shape. Higher AUC values indicate stronger neural responses across the given spatial-frequency range; lower values suggest weaker responses. Negative AUCs can occur if the area below the noise threshold exceeds the area above it.

#### Analysis of ssVEP data

Analysis of the preprocessed ssVEP data was conducted in python v3.9.18 (Python Software Foundation) using pingouin [32] and in R v4.4.1 [33] using lme4 [34], emmeans [35], and MKinfer [36], alongside standard python and R libraries. Family-wise error was controlled using the Bonferroni–Holm correction [37]. For analyses using bootstrapping or permutation testing, the number of iterations (1,000 or 100,000, depending on computational complexity of the analysis) was chosen pragmatically to balance computational feasibility with precision.

##### Comparisons of overall ssVEP response

Overall ssVEP responses, quantified by ssVEP AUC, were compared among *CRB1* subgroups and sighted controls using a linear mixed model (LMM) with group included as a fixed effect. Random intercepts per participant were included to account for interindividual heterogeneity and the inclusion of 2 eyes per participant. Post hoc tests assessing group differences were conducted using pairwise comparisons of the groups’ estimated marginal means derived from our LMM. To ensure the robustness of our findings, we replicated our comparisons using exact permutation tests estimated by Monte Carlo (100,000 replications).

##### Comparisons of the ssVEP curve shape

We investigated ssVEP curve shape differences by fitting a log parabola to the raw SNR data from each group using the equation outlined in Farahbakhsh et al. [20] and related work [38]. This model, originally developed to characterize contrast sensitivity across different spatial frequencies, provides a concise and theoretically grounded description of the ssVEP turning curve using 3 easily interpretable parameters [39]: *F*_max_, the spatial frequency at which SNR is maximal; *G*_max_, the curve’s peak gain (corresponding to SNR at *F*_max_); and *β*, the high-frequency falloff (full-width half maximum, in octaves). Curves were fitted using a maximum-likelihood estimation algorithm following a multistart optimization approach. Parameter uncertainty was estimated via participant-level bootstrapping (1,000 resamples), with the standard deviation of the resampled parameter estimates reported as the bootstrap standard error.

We assessed group differences in these parameters via pairwise permutation testing. For each group comparison, group assignment was randomly shuffled, creating 2 new, simulated datasets. The log parabola was fitted to each simulated dataset using the approach outlined above, and group differences were calculated for each parameter. This procedure was repeated 1,000 times to simulate a null distribution, against which the observed (real) group differences were compared to obtain *P* values [37].

##### Validity and reliability

We assessed the validity of our EEG-based measure by conducting linear mixed-model and Pearson correlation analyses between ssVEP AUC and both visual acuity and peripheral visual field loss. To confirm that our analyses were not confounded by age effects unrelated to vision loss severity, we modeled the effect of age on ssVEP AUC in sighted controls using a separate linear mixed model. Categorical acuity entries (hand movement, HM; PL) were converted to logMAR equivalents for this analysis following commonly applied clinical conventions, with HM assigned 2.3 logMAR and PL assigned 2.7 logMAR [3,40]. As these values do not represent true interval-scaled measures, analyses including them should always be interpreted with caution. To verify the robustness of our findings, we therefore replicated all analyses after exclusion of categorical acuity entries. As shown in Fig. S3, our findings remain consistent even when eyes with categorical visual acuity entries are excluded from our analyses.

To evaluate the reliability of our measure, we used 2 complimentary approaches. We used a resampling-based reliability assessment to evaluate the consistency of our measure across individual stimulation segments, preserving the data structure and analysis procedure used to derive our original measure, and a split-half approach that evaluates intrasession test–retest reliability across 2 repeated identical runs of our paradigm, separated by a short rest break, to emulate a repeated testing design.

For the resampling-based reliability assessment, we generated 1,000 bootstrapped datasets per tested eye by randomly sampling individual segments with replacement from the epoched EEG data while maintaining the original segment count (20 segments per spatial frequency and eye). For each bootstrapped dataset, the PDS, ssVEP SNR curve, and the ssVEP AUC were derived as previously outlined. We then computed the intraclass correlation (ICC) coefficients across the 1,000 resampled outcome measures using 2-way random-effects models (ICC(2); “single raters”) to evaluate the reliability (agreement) across resamples.

To obtain split-half test–retest reliability, our outcome measures were calculated separately across epochs from a first and second identical run (10 segments per spatial frequency each) separated by a short break (see Fig. 2). We correlated the resulting ssVEP data between the 2 runs and conducted permutation paired *t* tests to assess whether ssVEP responses differed systematically.

## Results

### ssVEP characteristics of *CRB1* phenotypes

**Fig. 4.**
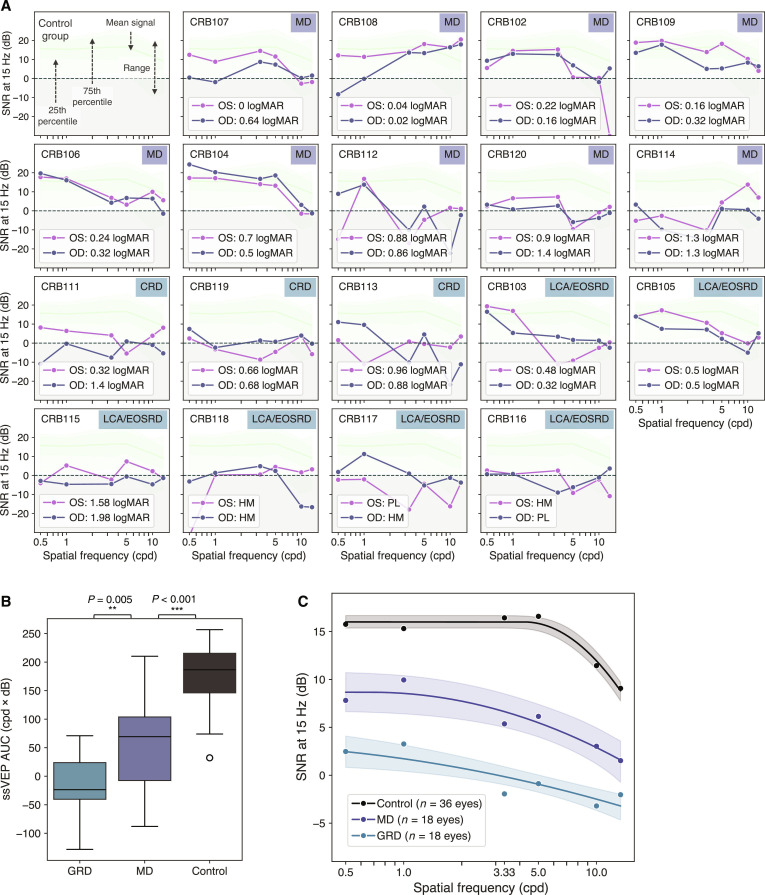
ssVEP curves for patients with *CRB1* retinopathy and subgroup comparisons. (A) SNR at the 15 Hz phase-reversal frequency of for the left (OS) and right eye (OD) of *CRB1* patients across different spatial frequencies, ordered by phenotype and visual acuity. The mean signal of the control group (*n* = 18), along with the 25th/75th percentile and the full range, is depicted in green and light green, respectively, for reference. The dotted gray area represents the noise floor where signal ≤ noise. (B) Comparison of ssVEP responses to pattern-reversing sinusoidal gratings among *CRB1* subgroups and controls for ssVEP AUC. (C) Average ssVEP responses and fitted log parabola per group. Shaded confidence bands represent ±1 standard deviation derived from bootstrapping (1,000 resamples of the original data with refitted curves). CRD, cone-rod dystrophy; GRD, generalized retinal degeneration; LCA/EOSRD, Leber congenital amaurosis/early-onset severe retinal degeneration; MD, macular dystrophy. ***P < 0.001, corrected; **P < 0.01, corrected.

Patients with *CRB1*-related retinopathy display distinct ssVEP curves, each reflecting the unique responsiveness of their retinocortical pathways to different spatial frequencies (Fig. 4A). Decreased visual acuity generally corresponds to lower ssVEP SNR; nearly all patients fall below the average control response for most spatial-frequency conditions. Patients with asymmetric vision typically exhibit attenuated ssVEP SNR with signal drop-off at lower spatial frequencies in the worse compared to the better eye. Patients with the LCA/EOSRD or CRD phenotype show particularly low SNR across spatial frequencies, with responses frequently falling below the noise threshold.

A key aim of our study was to systematically investigate ssVEP characteristics across phenotypes with varying retinal degeneration and vision levels. *CRB1*-related retinopathy is typically classified into LCA/EOSRD, CRD, MD, and RP, but due to rare disease status, we had no access to individuals with RP and only 3 patients with the CRD phenotype, a subgroup too small to be analyzed independently. The included CRD patients were older (*M*_age_ = 40.9 y) and were displaying advanced disease (*M*_disease duration_ = 22.2 y) involving macular-to-peripheral retinal degeneration with substantial impact on visual acuity and overall function. For group analyses, they were therefore integrated with the LCA/EOSRD group, who share the feature of generalized retinal degeneration. Figure S4 presents ssVEP responses for the MD, CRD, and LCA/EOSRD phenotypes, illustrating that responses in CRD patients closely resembled those of the LCA/EOSRD group. Crucially, as shown in Figs. S4 to S8 and Table S4, excluding CRD patients from our analyses did not alter our results.

Our grouping strategy, thus, gave rise to 2 *CRB1* groups of 9 patients each, one presenting with localized MD and one with generalized retinal degeneration, comprising those with LCA/EOSRD or advanced CRD. The 2 groups did not significantly differ in age or sex (see Table S1). However, generalized retinal degeneration participants had significantly worse visual acuity (*M* ± SD: 1.38 ± 0.88 logMAR vs 0.55 ± 0.46 logMAR; *P* = 0.001) and longer disease duration (*M* ± SD: 26.07 ± 14.52 y vs 14.52 ± 9.16 y; *P* = 0.039) compared to the MD group.

Group-level analysis confirmed the patterns identified on the single-subject level, revealing significant differences in overall ssVEP responses between all subgroups. A type III ANOVA on the LMM comparing ssVEP AUC between subgroups confirmed a significant main effect of group (*F*(2, 36) = 42.12, *P* <0.001, partial *η*^2^ = 0.70, and *R*^2^_marginal_ = 0.65), with subsequent pairwise comparisons of estimated marginal means indicating that ssVEP AUC was lowest in those with generalized retinal degeneration, intermediate in MD, and highest in controls (see Fig. 4B and Table S2). Permutation tests confirmed all group differences (see Table S3).

These pathology-related differences were mirrored in ssVEP tuning curve parameters: Compared to controls (*G*_max Controls_ = 16, bootstrap standard error [SE] = 0.65), both patient groups showed significantly attenuated peak response amplitudes (*G*_max GRD_ = 3.26, bootstrap SE = 2.08; *G*_max MD_ = 8.67, bootstrap SE = 2.05; both *P* < 0.001). Patients further showed significant changes in their curve shape (see Fig. 4C), with ssVEP responses peaking at significantly lower spatial frequencies (*F*_max GRD_ = 0.08, bootstrap SE = 0.92; *F*_max MD_ = 0.68, bootstrap SE = 2.32; *F*_max Control_ = 4.20, bootstrap SE = 0.84; *P*_GRD vs Control_ = 0.021, *P*_MD vs Control_ = 0.035) and declining at a more gradual rate at higher spatial frequencies (*β*_GRD_ = 4.43, bootstrap SE = 2.82; *β*_MD_ = 1.71, bootstrap SE = 1.45; *β*_Control_ = 0.80, bootstrap SE = 0.06; *P*_GRD vs Control_ = 0.024, *P*_MD vs Control_ = 0.035). These changes were most pronounced in the generalized retinal degeneration group. However, curve shape differences between *CRB1* subgroups did not reach significance after correcting for multiple comparisons (*P* = 0.311), suggesting that AUC may provide a more robust indicator of group membership.

### Clinical relevance and validity—correlation with visual acuity and peripheral visual field loss

Next, we explored the relationship of our EEG-based measure with the current gold-standard visual acuity measure, which primarily evaluates foveal visual function, and a measure of peripheral visual field loss assessed using kinetic perimetry. This served multiple purposes: first, to validate the clinical relevance of our measurements; second, to elucidate the link between visual behavior and neuronal responsivity to visual stimuli in the visual cortex; and third, to gain insight into how pathology in different parts of the visual field influences neural activity at the visual cortex detected by EEG recordings.

**Fig. 5.**
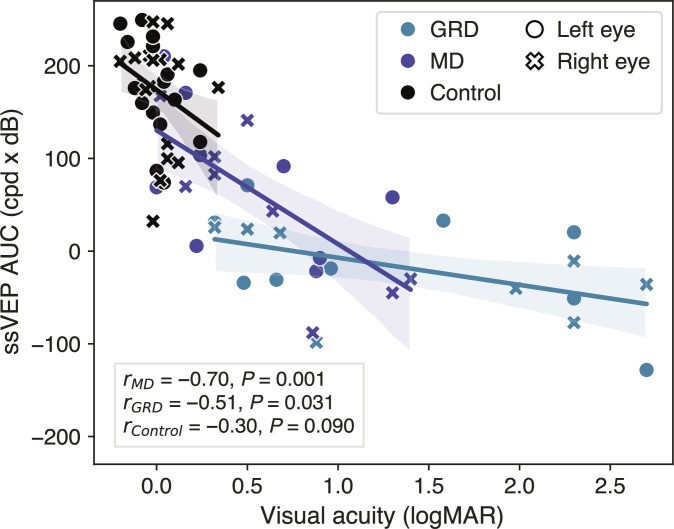
Correlation between ssVEP AUC and visual acuity for the two CRB1 groups and the control group respectively. AUC, Area under the curve; Cpd, cycles per degree; GRD, Generalised Retinal Degeneration; MD, Macular Dystrophy

First, we assessed the total observed association between ssVEP AUC and visual acuity at the group level using Pearson correlations for descriptive purposes. Across all participants, ssVEP AUC was strongly correlated with visual acuity (*r* = -0.75, *P* < 0.001), revealing that better letter acuity, determined through behavioral testing, was associated with increased neural responsivity of visual cortex neurons, as measured using occipital scalp EEG. This correlation persisted within the 2 *CRB1* groups separately but not the control group (see Fig. 5), which was expected as the presented stimuli were deliberately chosen to fall below the thresholds of typically sighted individuals [19] to optimize sensitivity in the low-vision range.

To account for the nonindependence of 2 eyes per participant, we confirmed this relationship using a linear mixed-effects model. The model showed a significant association between ssVEP AUC and visual acuity across the whole sample (*β* = −0.002 ± 0.0007, *P* = 0.003) and within the *CRB1* groups (*β*_GRD_ = −0.004 ± 0.001, *P* = 0.002; *β*_MD_ = −0.003 ± 0.001, *P* = 0.036), but not in controls (*β*_Control_ = 0.0002 ± 0.001, *P* = 0.848). In other words, patients whose acuity differed by approximately 0.3 logMAR—equivalent to a 3-line difference on a standard letter chart—showed an ssVEP difference in the order of 100 AUC units. We found no significant effect of age on ssVEP AUC in our control group (*β* = 0.17 ± 1.17, *P* = 0.889), indicating that these effects are unlikely to be driven by age-related differences independent of vision loss.

We found no significant correlation between SNR AUC and indices of the peripheral visual field border (see Fig. S1). This could be due to a lack of statistical power. However, it is also consistent with evidence that ssVEPs are more influenced by central visual field activity, with neurons encoding the far periphery close to the outer visual field boundary—located deeper in the brain—contributing less to the EEG signal [41,42].

### Reliability of ssVEP assessments across *CRB1* phenotypes

To investigate the reliability of our main ssVEP outcome measure, we performed a resampling-based reliability assessment on 1,000 bootstrapped datasets per tested eye and calculated the ICC, with values close to 1 indicating high reliability and those close to zero indicating low reliability. The ICC on the resampled dataset indicated excellent absolute agreement across bootstrapped ssVEP AUC data (*ICC*(2) = 0.95; 95% confidence interval [0.93, 0.96]; *P* < 0.001).

Next, we assessed intrasession retest reliability by computing the ssVEP AUC separately for the first and second runs of our experimental procedure and comparing the resulting values. This analysis emulates the reliability of ssVEPs under a test–retest scenario but is less representative of our earlier reported measures as only half of the data are considered. Again, reliability was high; the ssVEP AUCs in run 1 and run 2 were strongly correlated (*r* = 0.89, *P* < 0.001) across the whole sample. Permutation paired *t* tests indicated no significant difference between the ssVEP AUC of the 2 runs (permutation mean difference = 4.87, SE = 5.03, 95% confidence interval [−5.65, 15.37], *P* = 0.354), indicating no significant learning or fatigue effects. This is in line with the passive nature and tolerability of the task.

**Fig. 6.**
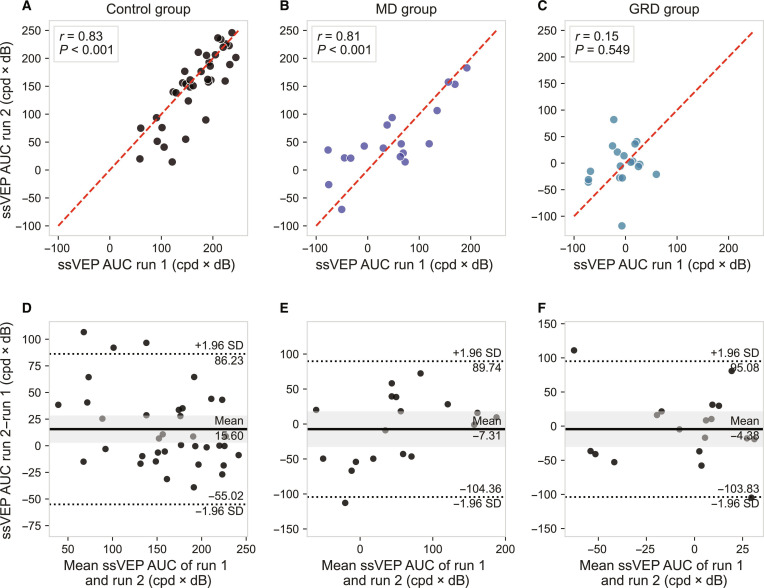
Intrasession reliability for ssVEP AUC for the control, MD, and GRD group, respectively. (A to C) Correlation between ssVEP AUC calculated for the first and second runs. Each data point represents the ssVEP AUC for one eye in response to one stimulus condition. (D to F) Bland–Altman plots for ssVEP AUC calculated separately for the first and second runs.

These high reliabilities were consistent when the control and the MD group were analyzed separately. However, in the generalized retinal degeneration group, the reliability of ssVEP responses to sinusoidal gratings was notably poor. This is illustrated in Fig. 6, which presents run-to-run correlations and Bland–Altman plots for the ssVEP AUC. Further analysis revealed that this poor reliability in patients with generalized retinal degeneration was primarily driven by a high proportion of ssVEP responses where the signal fell below the noise floor (SNR < 0), reflecting the severity of their visual impairment. It is noteworthy that despite this, the correlation with acuity was still present, meaning that this noisier signal still carries substantial and functionally relevant information.

**Fig. 7.**
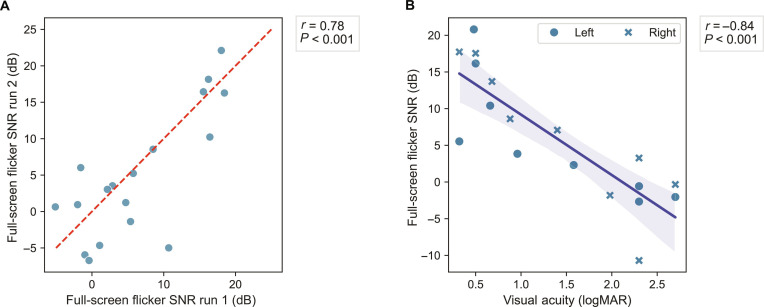
Analysis of full-screen flicker ssVEP in the generalized retinal degeneration group. (A) Intrasession reliability calculated as the correlation between run 1 and run 2. (B) Correlation with visual acuity for the generalized retinal degeneration group.

Nevertheless, to improve the sensitivity of our measure in the lowest-vision *CRB1* patients, we evaluated the reliability and clinical utility of a full-screen flicker ssVEP stimulus, purposefully interspersed with stimuli with spatial components in our paradigm to induce signal in low vision. Indeed, full-screen flicker stimuli demonstrated good reliability in the generalized retinal degeneration group (see Fig. 7A), and full-screen flicker SNR also strongly correlated with visual acuity (see Fig. 7B). This highlights the value of this stimulus for objectively quantifying visual function in the very-low-vision range. Importantly, however, while full-screen flicker was more effective in assessing vision in severe impairment, it did not differentiate well between the *CRB1* phenotypes (see Fig. S2), potentially reflecting saturation of the ssVEP signal for full-field luminance variations when a degree of function is present. This highlights full-field stimulation as a useful complement to ssVEP pattern stimuli when characterizing function across diverse phenotypes.

## Discussion

We characterized ssVEP profiles in individuals with molecularly confirmed *CRB1*-related retinopathy, a condition marked by a strikingly broad spectrum of clinical phenotypes, whose VEP profiles have to date not been systematically characterized. By employing a large-field ssVEP paradigm using both pattern-reversal and full-screen flicker stimuli, we aimed to address the challenge of objectively characterizing visual function in this heterogeneous patient population.

Our results revealed a significant decrease in amplitude across the ssVEP curve among *CRB1* patients, most pronounced in those with generalized retinal involvement (LCA/EOSRD and advanced-stage CRD phenotype) and milder, yet still significant, in those with MD. ssVEP responses thus not only distinguished *CRB1* patients from controls but also differentiated between *CRB1* subgroups with distinct retinal characteristics and vision levels.

Patients also exhibited significant changes in tuning curve shape. Specifically, their preferred spatial frequency (*F*_max_) was shifted to lower values, and they showed a more gradual decline of ssVEP signal at higher frequencies. There are several possible explanations for this shift. First, as central input deteriorates, ssVEP signals could undergo a relative upweighting of more peripheral field contributions, which show decreased gain and lower preferred spatial frequencies compared to more central locations [43–46]. Second, the disease might also change the functional characteristics of the surviving macular tissue itself. For example, in *CRB1*-related retinopathies, altered retinal lamination and underdeveloped foveal regions [3] might modify how macular circuits process spatial information at the retinal level and induce shifts in spatial-frequency tuning that propagate to later visual processing stages. Finally, this shift in ssVEP curve shape could also be explained by oculomotor factors: High spatial-frequency patterns are disproportionally affected by fixation instability and nystagmus, as even small eye movements can blur their representation on the retina and reduce retinocortical signal strength. At this stage, these interpretations are speculative and clarifying them will require studies with spatially precise measures of retinocortical signaling, such as confocal EEG or high-resolution fMRI.

### ssVEPs as an indicator of functional vision

Critically, ssVEP responses strongly correlated with visual acuity across *CRB1* subgroups. This highlights that both measures carry meaningful information. As visual acuity is one of the gold-standard outcome measures of clinical trials, the fact that we obtained high correlations with ssVEP response even in our low-vision group is notable and validates ssVEPs as a meaningful index of visual function in this hard-to-characterize population. In controls, there was no correlation between acuity and ssVEPs, which is expected as our stimulus range, optimized to detect differences among vision loss patients, was designed to fall below the range required to detect acuity variations in those without eye disease [19]. Nevertheless, previous work has shown that comparable methods can sensitively capture visual acuity in normally sighted participants when higher spatial frequencies are used [19].

ssVEPs did not correlate with the outer peripheral visual field boundary, which could reflect relative insensitivity of scalp-recorded VEPs to the very far periphery [41,42]. Central visual field metrics might relate more closely to ssVEP responses; however, such measures are challenging to obtain reliably across the full spectrum of visual function and were not available in our cohort. Alternatively, our result could also be explained by limited statistical power or underrepresentation of peripheral visual field loss. *CRB1* mutations can also manifest as RP, characterized by rod-dominant peripheral retinal degeneration—a phenotype not represented in our study. Including this phenotype within a larger, more diverse sample and using comprehensive measures of visual field function could clarify the relative contribution of central and peripheral loss to ssVEP responses and whether central field measures could capture variance in ssVEPs beyond that explained by visual acuity.

### Reliability of ssVEP measures and the role of full-screen flicker stimuli

Two complementary reliability-analysis methods confirmed good reliability of ssVEP curve measures in the control and MD groups. In patients with generalized retinal degeneration, however, the reliability of these measures was poor, which is consistent with their severe visual impairment and perceptual experience during the experiment. For many participants in this group, most gratings were too fine for their visual system to pick up. As a result, participants often reported seeing no discernible pattern and ssVEP responses to these stimuli fell near or below the noise threshold. In these cases, the measured EEG signal is dominated by random noise, which does not correlate strongly across trials, explaining the low signal reliability in these cases. Despite this, the correlation with visual acuity persisted in this group, indicating that despite these noisier signals, ssVEP AUC retains functionally relevant information.

The inclusion of a full-screen flicker stimulus in our paradigm allowed us to induce more reliable ssVEP signals in the generalized retinal degeneration group, which also strongly correlated with visual acuity, highlighting its value for objectively capturing visual function in the very-low-vision range. In line with this, we recently used the full-screen flicker stimulus to demonstrate significant rescue of function in toddlers with severe *AIPL1*-linked LCA after gene therapy [47]. While this stimulus was particularly effective in quantifying vision in cases of severe impairment, it was less effective at differentiating between *CRB1* subgroups, likely due to signal saturation at higher levels of visual function [48].

### Considerations for clinical trials

Our results highlight several considerations for those aiming to objectively capture visual function in treatment trials. When assessing visual function across a broad spectrum of abilities, it is beneficial to incorporate both pattern-reversal and full-screen flicker stimuli as complementary components within a single ssVEP testing session. This is especially useful for longitudinal studies where patients may vary in baseline vision and can move meaningfully within and between functional ranges, and where it is hard to predict the extent of change across all individuals in the cohort. Within this framework, the stimulus spectrum can also be tailored to baseline function and expected change to reduce testing time. However, adding both stimulus types offers substantial benefits. It increases the chance of a reliable baseline, even for individuals with low vision. It also allows for vision stratification of end points within the cohort, where full-screen flicker ssVEP provides the most reliable readout in very-low-vision phenotypes, while pattern ssVEP AUC is more informative for focal loss and earlier disease stages. Shifts in the ssVEP profile across stimulus types further provide important information on clinically meaningful change: flicker ssVEPs emerging from previously subthreshold responses indicate the emergence of cortical light sensitivity, while newly detectable pattern ssVEPs suggest the emergence of patterned vision. Clinically meaningful change in longitudinal ssVEPs can also be expressed in terms of associated acuity gain. For example, we show that across patients, a 100-unit increase in pattern ssVEP AUC corresponds roughly to a 3-line difference in a standard letter chart, a standard benchmark of meaningful change in clinical trials. However, as these values are based on between-subject comparisons, they require longitudinal validation.

We consider ssVEPs a useful supportive biomarker of visual system function for clinical trials due to their association with behavioral vision measures and their ability to provide mechanistic explanations of change when combined with biologically informed modeling techniques. However, in contexts where behavioral measures can be difficult or impossible to obtain reliably, such as in very young children, ssVEPs can also serve as secondary endpoints, providing objective assessment of visual function that complements and supports behavioral tests of treatment efficacy—see for example the use of this measure in *AIPL1*-linked gene therapy [47]. In early-phase studies, ssVEPs could further function as an exploratory pharmacodynamic marker, capturing neural-level effects that may precede perceptual improvements.

Finally, in clinical research, the nonindependence of 2 eyes within each participant adds an important layer of complexity that should not be ignored when interpreting functional outcomes. Analyses that average eyes or treat them as independent units risk losing power, obscuring meaningful within-subject variability, or introducing bias. This is particularly relevant in conditions like *CRB1*-related retinopathy, where retinal dystrophy can be asymmetric, and in monocular treatment trials, where therapeutic benefit is expected in only one eye. Mixed-effects models, as used in this study, allow for robust statistical comparisons while correctly modeling within-subject variability. They therefore offer a flexible modeling approach for future trials that can be extended to include additional hierarchical levels (e.g., repeated measures over time) or random slopes to model subject-specific longitudinal change.

### Limitations due to sample characteristics

While our study set out to characterize the full spectrum of *CRB1* retinopathy, limited patient numbers due to rare disease incidence prevented systematic comparison of all associated phenotypes. For group analyses, we therefore chose to combine patients with advanced-stage CRD (*n* = 3) and those with LCA/EOSRD (*n* = 6) based on their shared feature of generalized retinal involvement. This function-based grouping strategy aligns more closely with functional staging approaches increasingly used in clinical trials than classical phenotype-pure grouping. Although this is arguably not ideal from a natural history perspective, as it does not fully reflect the distinct nature of these phenotypes, it allowed us to compare ssVEP profiles between patients with localized MD and those with more widespread retinal involvement.

This highlights the inherent complexity of *CRB1*-phenotype classification, with overlapping symptoms, potential transitions between phenotypes over the course of the disease, and phenotypic distinctions that are often clearest in early stages. The extent of retinal involvement can vary considerably within a phenotype depending on disease progression, and phenotypes tend to converge functionally as degeneration advances, which complicates differentiation. In our sample, the LCA/EOSRD group spanned a wide range of visual function from mild visual impairment to no light perception, again depending on the stage of the disease, placing advanced-stage CRD patients in an intermediate position within this group in terms of acuity. This may differ in other cohorts, where it might be more appropriate to group CRD patients with MD. Based on typical degeneration patterns associated with each phenotype, we speculate that future studies with larger samples will find ssVEP responses from earlier-stage CRD patients falling between those of MD and EOSRD/LCA patients. Notably, excluding the CRD patients from these analyses did not affect our main findings (see Figs. S4 to S8 and Table S4).

### Strengths and limitations of our ssVEP measure

VEP assessments capture visual signal detection directly at the cortical level while patients watch movies interleaved with the visual stimuli of interest. This makes them response free and patient friendly, offering unbiased insight into visual function while improving comfort, enhancing attention to the screen, and minimizing frustration, especially in those with very low vision. These qualities also make ssVEPs well suited for pediatric populations, as we have demonstrated recently in young children undergoing *AIPL1* gene therapy, where this measure provided key objective evidence of functional rescue after treatment [47]. Assessing visual function at this intermediate level of processing further offers a valuable link between retinal integrity and behavioral vision assessments relying on subjective patient reports.

However, since VEPs aggregate activity across multiple visual processing stages, isolating contributions from individual stages is difficult, limiting the measure’s specificity. For instance, *CRB1* patients often present with nystagmus and roving eye movements, which can interfere with ssVEP signal strength [49], making it difficult to differentiate between signal changes due to retinal dysfunction and signal changes due to fixation instability. To complicate matters further, patients with more severe visual impairment also tend to struggle more with fixation, which we observed in our cohort through online video recordings during stimulation. Correcting for fixation instability using eye-tracking data or ocular electrode measurements could help isolate retinal function, but this is not trivial given the complex relationship between large eye movements and ssVEP signals and falls beyond the scope of this report. This limitation is particularly relevant when assessing changes in visual function following ocular treatments, as it is unclear to which degree improvements reflect better fixation rather than genuine changes in retinal function. Changes in fixation stability could also increase within-subject variance of ssVEP signals longitudinally, making it more difficult to detect small treatment-related effects to retinocortical function. Nonetheless, since fixation stability itself is an essential component of functional vision [50], a composite measure of improved fixation and visual computation that correlates strongly with acuity can offer a meaningful clinical outcome.

We selected ssVEPs for their robustness across populations, especially in children [51,52], and their reduced susceptibility to developmental effects [53] and employed large-field stimulus presentation to minimize the need for fixation [54]. However, as previously shown, pattern-reversal ssVEP paradigms are also particularly susceptible to the degrading effect of fixation instability on VEPs [49,55], so it is likely that further optimization of the stimulus parameters, such as using on/off stimuli, can achieve even better results. We chose sinusoidal gratings as our stimuli because they contain a single, well-defined spatial frequency [19], which makes them relatable to other gold-standard psychophysiological measures of visual function [15,56–58]. Previous work has further suggested an advantage of horizontal-bar stimuli over checkerboards in children with nystagmus [51]. Nonetheless, checkerboard stimuli are more commonly used in clinical practice and could be integrated into this approach to offer a more direct point of comparison with clinical standards. In our paradigm, pattern ssVEPs were presented in a fixed order within each stimulus cycle, consistent with the International Society for Clinical Electrophysiology of Vision standards for VEP assessments and with evidence that coarse-to-fine sequences can support attentional engagement in low-vision patients [26]. Prior work suggests that VEP-based vision assessments are relatively robust to presentation order [26], and we found no evidence of disproportionate time-on-task or fatigue effects across spatial frequencies (see Fig. S9). Nonetheless, potential order effects cannot be fully ruled out, and future studies should explore alternative presentation sequences to confirm the robustness of tuning curve parameters. Although our approach demonstrated clear effects, other VEP paradigms, particularly those designed to mitigate fixation instability, could thus potentially provide even more refined and accurate insight into visual processing in IRDs and improve signal in participants with very low vision.

## Conclusion

This study provides the first systematic characterization of ssVEP profiles in individuals with *CRB1*-related retinopathy, a condition associated with a remarkably wide range of clinical phenotypes. By combining pattern-reversal and full-screen flicker stimuli in our paradigm, we were able to obtain reliable visual function measures across a broad spectrum of visual abilities, including severe vision loss. Our findings highlight the utility of ssVEPs as an objective, noninvasive, and patient-friendly tool that complements traditional vision assessments, offering critical insights into disease characteristics and treatment outcomes. Importantly, due to its diverse phenotypic presentation, *CRB1*-related retinopathy can be viewed as a representative model for the broader group of IRDs, making our findings applicable to a wide range of related conditions. Future research should expand this approach to include additional phenotypes and refine paradigms for greater specificity and clinical impact.

## Data Availability

Due to the sensitive nature of the data, raw EEG data cannot be shared, but fully anonymized preprocessed data are available on Zenodo (https://doi.org/10.5281/zenodo.18956093). Code to reproduce the results reported in this paper are available at https://github.com/ChildVisionLab/crb1_ssvep_workflow, and code used for stimulus presentation are available at https://github.com/ChildVisionLab/EEG_Stimulation_CRB1.
