# Supplementary material for: Using Steady-State Visual Evoked Potentials to Characterize Wide-Ranging Retinopathy Linked to CRB1: Implications for Clinical Trials
Source: Comput Struct Biotechnol J. 2026 Apr 30;35(1):0042. doi: 10.34133/csbj.0042 (PMC13132498; doi:10.34133/csbj.0042)
Supplement: Supplementary 1 — Tables S1 to S4 Figs. S1 to S9 [file csbj.0042.f1.pdf]

Supplementary Material

Supplementary Material 1: Participant Demographics

Supplementary Table 1. Demographics *CRB1* subgroups and controls						
	**Control**	***CRB1*** **Macular Dystrophy**	***CRB1*** **Generalised retinal dystrophy**	$p_{Control\ vs\ MD}$	$p_{Control\ vs\ GRD}$	$p_{MD\ vs\ GRD}$
**n**	18	9	9	---	---	---
**Age**[1]	31.13 (10.93)	38.19 (15.28)	34.14 (14.52)	0.186	0.543	0.565
**Female/ Male**[2]	11/7	3/6	3/6	0.173	0.173	1
**Disease duration (years)**[1]	*NA*	14.52 (9.16)	26.07 (14.52)	---	---	**0.039**
**BCVA (logMAR)**[1]	0.01 (0.12)	0.55 (0.46)	1.38 (0.88)	**<0.001**	**<0.001**	**0.001**

[1]Mean (SD); Exact Permutation Test Estimated by Monte Carlo, 100'000 replications, for numeric variables with deviation from normal distribution and/or equal variances
[2]Chi-squared test for categorical variables

Supplementary Material 2: Expanded Overview of Main Statistical Comparisons and Replications Using Non-Parametric Tests

Supplementary Table 2. Pairwise Comparisons of ssVEP AUC Using Contrasts on the Previously Computed Linear Mixed Model

Comparison	EMM diff	SE	df	t-ratio	effect size	p
Control vs Generalised Retinal Dystrophy	194.82	23.00	39.3	8.49	4.62	**<0.001**
Control vs Macular Dystrophy	114.12	23.00	39.3	4.97	2.71	**<0.001**
Macular vs Generalised Retinal Dystrophy	80.70	26.50	39.3	3.05	1.91	**0.004**

EMM diff, estimated marginal means difference; effect size, Cohen effect size of estimated marginal means difference; SE, standard error; df, degrees of freedom; p-values were corrected for multiple comparisons using the Bonferroni-Holm method.

Supplementary Table 3. Pairwise Comparisons of ssVEP AUC using Permutation Tests

Comparison	Mean difference	p	Cohen's d
Control vs Generalised Retinal Dystrophy	194.82	**<0.001**	3.59
Control vs Macular Dystrophy	114.12	**<0.001**	1.75
Macular vs Generalised Retinal Dystrophy	80.7	**0.001**	1.22

Note: To ensure the robustness of our findings, we replicated our previous group comparisons of ssVEP AUC using exact permutation tests estimated by Monte Carlo (100'000 replications). p-values were corrected for multiple comparisons using the Bonferroni-Holm method.

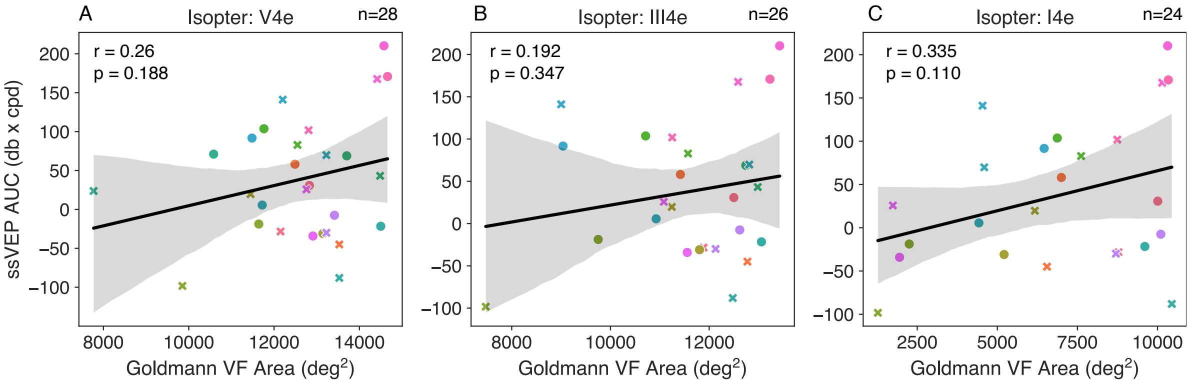

***Supplementary Figure 1*. Correlation between ssVEP AUC and kinetic visual field measures in the *CRB1* group.**

A) Correlation with peripheral visual field loss assessed using a V4e isopter (1.7° diameter; 64 mm^2 area) (Racette et al., 2019) **B)** Correlation with peripheral visual field loss assessed using a III4e isopter (0.8° diameter; 4 mm^2 area). **C)** Correlation with peripheral visual field loss assessed using a I4e isopter (0.1° diameter; 0.25 mm^2 area). Colours indicate participant ID; shape indicates left vs. right eye. All stimuli used the maximal stimulus intensity 4e.

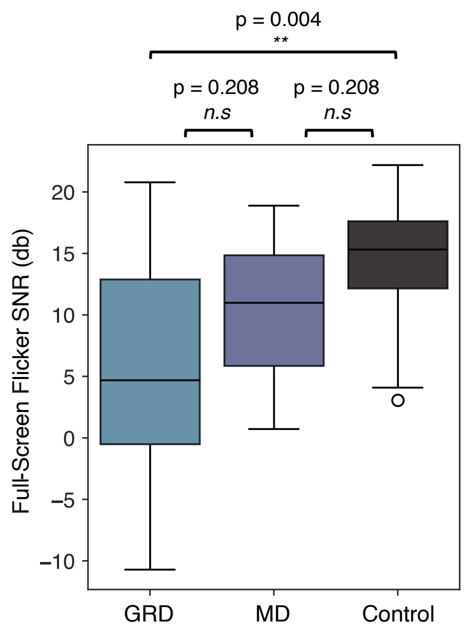

Supplementary Figure 2.
Comparison of ssVEP responses to full-screen flashing stimuli among *CRB1* subgroups and controls

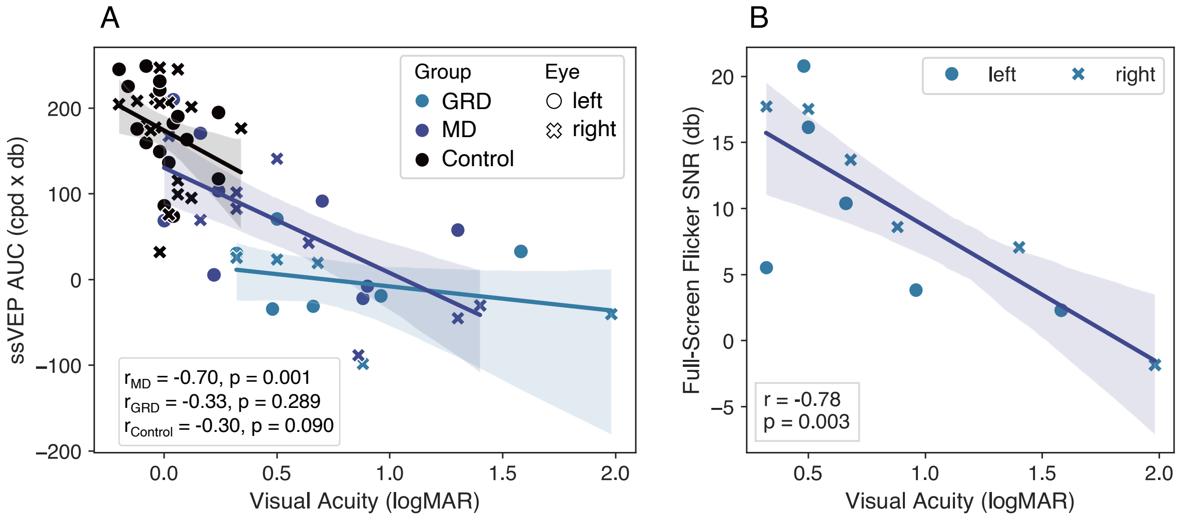

***Supplementary Figure 3.* Replication of analyses after exclusion of eyes with categorical visual acuity entries**

A) Pearson correlation between ssVEP AUC and visual acuity for the two *CRB1* subgroups and the control group respectively. Across all participants, ssVEP AUC was strongly correlated with visual acuity (r = -0.75, p < 0.001). This correlation persisted in the Macular Dystrophy (MD) subgroup. Note that exclusion of eyes with categorical acuity measures drastically reduces sample size of the generalised retinal dystrophy group. A linear mixed model indicated a significant association between ssVEP AUC and visual acuity in both patient subgroups ($\beta_{GRD} = -0.004 \pm 0.004$, *p* = .028, $\beta_{MD} = -0.003 \pm 0.0009$, *p* = .002, $\beta_{Control} = 0.0001 \pm 0.0007$, *p* = .8422). **B)** Pearson correlation between Full-field flicker ssVEP and ssVEP responses to full-screen flashing stimuli in the generalised retinal dystrophy group.

Supplementary Material 3: Replication of Data Analysis on the Phenotype Level

To investigate whether the previously identified subgroup differences were influenced by our grouping strategy, we reanalysed our data using the original phenotype classification. An overview of ssVEP profiles across these groups are provided in Supplementary Figure 4. Due to its small sample size, the CRD group (n=3) had to be excluded from further statistical analyses. Consequently, the replication was conducted across the LCA/EOSRD (n=6), MD (n=9), and sighted control group (n=18).

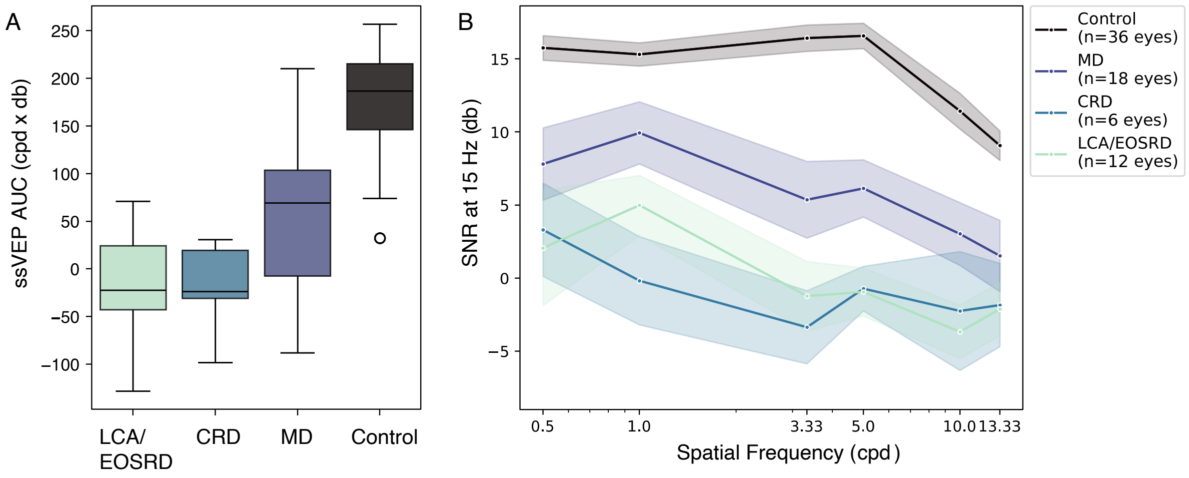

***Supplementary Figure 4*. ssVEP curves for patients with CRB1-retinopathy according to phenotype.**

A) ssVEP AUC for pattern-reversing sinusoidal gratings among *CRB1* phenotypes and controls
B) ssVEP responses and confidence intervals across spatial frequencies among *CRB1* phenotypes and controls

AUC, Area under the curve; Cpd, cycles per degree; CRD, Cone Rod Dystrophy; LCA/EOSRD, Leber Congenital Amaurosis/Early onset severe retinal dystrophy; MD, Macular dystrophy; SNR, signal-to-noise ratio

We first investigated differences in ssVEP AUC across these groups using a linear mixed model with Group included as a fixed effect and participant-specific random intercepts. Like in the main analysis, a type III ANOVA revealed a significant main effect of group ($F(2, 33)$ =31.79, p<0.001, partial η^2 = 0.66, $R^2_{marginal}$ = 0.60) and subsequent pairwise comparisons of estimated marginal means indicated that ssVEP AUC was lowest in the LCA/EOSRD phenotype, intermediate in MD, and highest in controls (see Supplementary Table 6).

Supplementary Table 4. Pairwise Comparisons of ssVEP AUC Using Contrasts on a Linear Mixed Model with Group Included as a Fixed Effect and Participant-Specific Random Intercepts						
**Comparison**	**EMM diff**	**SE**	**df**	**t-ratio**	**EMM eff (d)**	**p**
Control vs MD	114.12	27.5	36.3	7.03	4.62	**<0.001**
Control vs LCA/EOSRD	193.47	23.8	36.3	4.79	2.73	**<0.001**
MD vs LCA/EOSRD	79.35	30.8	36.3	2.58	1.9	**0.014**

EMM diff, estimated marginal means difference; EMM eff (d), effect size of estimated marginal means difference; SE, standard error; df, degrees of freedom; p-values were corrected for multiple comparisons using the Bonferroni-Holm method.

Exclusion of patients with CRD did not alter the overall pattern of ssVEP tuning curve differences. Relative to sighted controls, the LCA/EOSRD group showed significantly decreased peak response amplitudes ($G_{max\ LCA/EOSRD} = 4.90$, $G_{max\ Controls} = 16$, $p_{GRD} < 0.001$), a shift towards lower preferred spatial frequencies ($F_{max\ GRD} = 0.04$, $F_{max\ Control} = 4.20$, p = 0.017), and more gradual rate of decline at higher spatial frequencies ($\beta_{GRD} = 4.43$, $\beta_{Control}$ = , p = 0.027). Consistent with the main analysis, curve shape differences between the milder MD and the more severe LCA/EOSRD group did not reach significance after correcting for multiple comparisons (p = 0.286)

The correlation with visual acuity also remained largely unchanged after the exclusion of CRD subjects, showing moderate-to-strong correlations with ssVEP AUC in both the MD and LCA/EOSRD groups (see Supplementary Figure 5) that was confirmed in a linear mixed model analysis ($\beta_{EORD} = -0.003 \pm 0.001$, $p = .005$, $\beta_{MD} = -0.002 \pm 0.001$, $p = .019$, $\beta_{Control} = 0.0003 \pm 0.0008$, $p = .739$). We again found no significant correlation between ssVEP AUC and any indices of peripheral visual field extent (see Supplementary Figure 6).

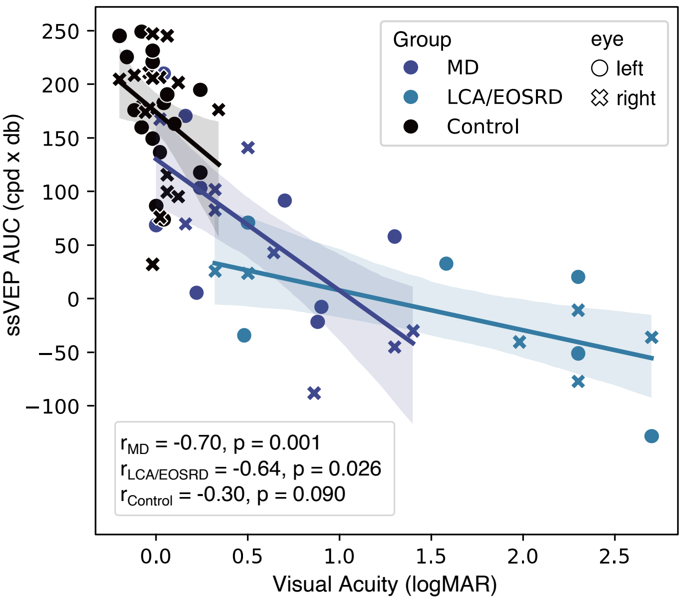

***Supplementary Figure 5.* Correlation between SNR (db) AUC and visual acuity for the two *CRB1* phenotypes and the control group respectively, after exclusion of the three *CRB1*-CRD patients**

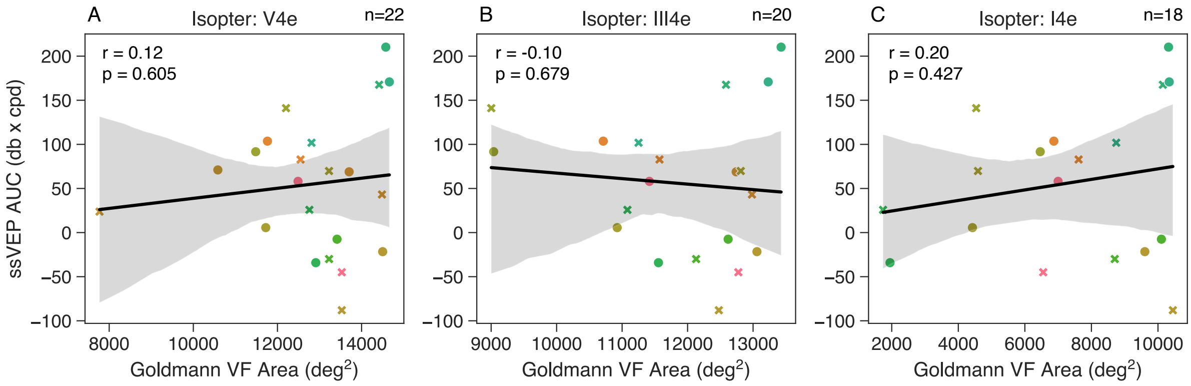

***Supplementary Figure 6.* Correlation between ssVEP AUC and kinetic visual field measures in the *CRB1* group after exclusion of the three *CRB1*-CRD patients.**

A) Correlation with peripheral visual field loss assessed using a V4e isopter (1.7° diameter; 64 mm^2 area) (Racette et al., 2019) **B)** Correlation with peripheral visual field loss assessed using a III4e isopter (0.8° diameter; 4 mm^2 area). **C)** Correlation with peripheral visual field loss assessed using a I4e isopter (0.1° diameter; 0.25 mm^2 area). Colours indicate participant ID; shape indicates left vs. right eye. All stimuli used the maximal stimulus intensity 4e.

The exclusion of the CRD groups had no meaningful impact on reliability estimates of our measure. A resampling-based reliability assessment on 1’000 bootstrapped datasets per tested eye dataset confirmed excellent absolute agreement across bootstrapped ssVEP AUC data (ICC(2) = 0.95; 95% CI: [0.93, 0.96]; $p < 0.001$).

Similarly, this did not significantly affect intra-session retest reliability estimates, as correlations between ssVEP AUC in the first and second run remained strong across the

full sample for both (r = .88, p<0.001). Permutation paired t-tests again indicated no significant difference between ssVEP AUC of the two runs (permutation mean difference = 4.88, SE = 5.03, CI = [-5.69, 15.41], p=0.357).

Since the excluded CRD subjects had low visual acuity and weak ssVEP responses to the presented sinusoidal gratings, their removal slightly increased intra-session reliability estimates in the LCA/EOSRD group. However, reliability in this subgroup remained poor, as shown in Supplementary Figure 7.

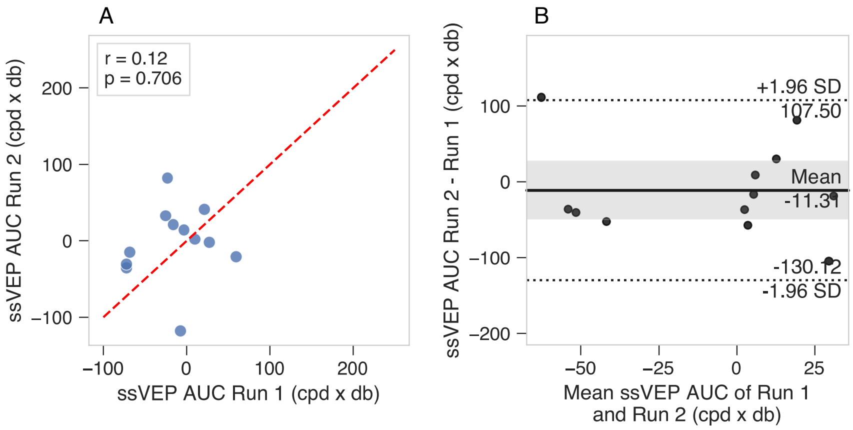

***Supplementary Figure 7*. Intrasession-reliability for ssVEP AUC and SNR per spatial frequency for the LCA/EOSRD group**
A) Correlation of ssVEP AUC between the first and second run. Each datapoint represents SNR AUC for one eye in response to one stimulus condition. **B)** Bland-Altman plot for ssVEP AUC calculated separately for the first and the second run

Similar to our results in the generalised retinal dystrophy group encompassing both LCA/EOSRD and CRD patients, full-screen flicker stimuli demonstrated good reliability in the LCA/EOSRD subgroup (see Supplementary Figure 8a), and full-screen flicker SNR strongly correlated with visual acuity (see Supplementary Figure 8b). Subsequent analyses further confirmed that while full-field flicker was more effective in assessing vision in severe impairment, it does not differentiate well between the *CRB1* phenotypes (see Supplementary Figure 8c). This confirms the role of full-field stimulation as a useful complement to ssVEP pattern stimuli when characterising visual function across diverse phenotypes.

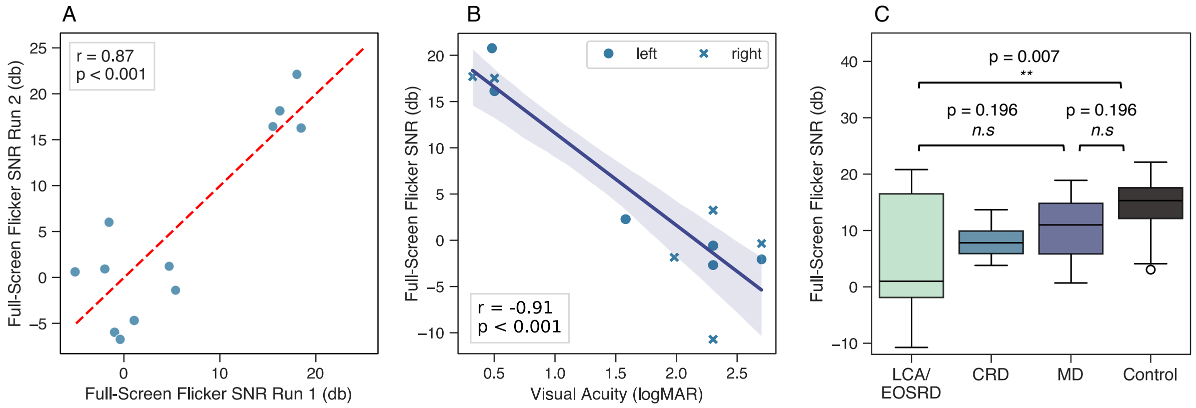

***Supplementary Figure 8*. Analysis of Full-field flicker ssVEP in the LCA/EOSRD group and phenotype comparison**

A) Intra-session reliability calculated as the correlation between run 1 and run 2 in the LCA/EOSRD group **B)** Correlation with visual acuity for the LCA/EOSRD group **C)** Comparison of ssVEP responses to full-screen flashing stimuli among *CRB1* phenotypes and controls. Due to the small sample size of the CRD group (n=3), this group could not be included in statistical group comparisons.

Supplementary Material 4: Investigation of Potential Order Effects

In our paradigm, pattern ssVEPs were presented in a fixed order within each stimulus cycle. To assess potential time-on-task or fatigue effects due to stimulus order, we examined whether responses for individual stimulus conditions changed disproportionately between runs. Visual inspection revealed no systematic order effect in the alignment of responses between runs (see Supplementary Figure 9). We then tested this formally using a linear mixed model, with spatial frequency and run included as fixed effects, a spatial frequency x run interaction, and random intercepts per participant. As expected, an ANOVA on this model indicated a significant effect of spatial frequency on ssVEP SNR ($F(1, 972) = 152.78$, $p<.001$), reflecting expected ssVEP amplitude differences across spatial frequencies. However, there was no significant difference across runs ($F(1, 972) = 0.62$, $p = .433$) or interaction between spatial frequency and run ($F(1, 972) = 0.01$, $p = .9145$), indicating that responses were stable across runs and that our main results were unlikely to be systematically influenced by stimulus order, fatigue, or time-on-task effects.

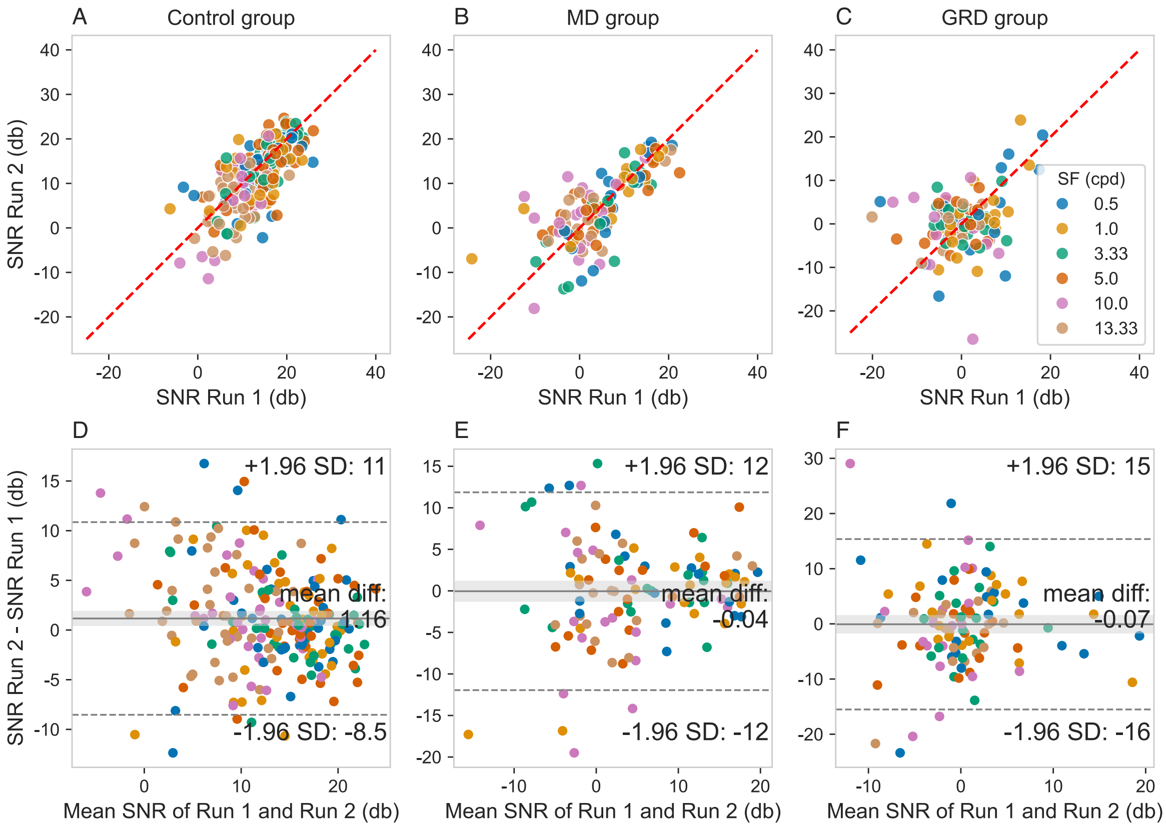

***Supplementary Figure 9*. Intrasession-reliability for ssVEP SNR for the control, Macular Dystrophy (MD), and generalised retinal degeneration (GRD) group per spatial frequency.**

A)-C) Correlation between ssVEP AUC calculated for the first and second run per spatial frequency. Each datapoint represents the ssVEP AUC for one eye in response to one stimulus condition. **D-F)** Bland-Altman plot for ssVEP SNR calculated separately for the first and second run per spatial frequency.
